# The immune tolerance role of Bregs in inhibiting human inflammatory diseases, with a focus on diabetes mellitus

**DOI:** 10.3389/fimmu.2025.1565158

**Published:** 2025-04-30

**Authors:** Qi Zhang, Jinfeng Liao, Zheng Liu, Siyuan Song, Limin Tian, Yi Wang

**Affiliations:** ^1^ Department of Laboratory Medicine, West China Second University Hospital, Sichuan University, Chengdu, Sichuan, China; ^2^ Key Laboratory of Birth Defects and Related Diseases of Women and Children, Sichuan University, Ministry of Education, Chengdu, Sichuan, China; ^3^ Department of Dermatology, Sichuan Provincial People’s Hospital, University of Electronic Science and Technology of China, Chengdu, Sichuan, China; ^4^ Pathology Department, University of Texas, MD Anderson Cancer Center, Texas, Houston, TX, United States; ^5^ Department of Neuroscience, Baylor College of Medicine, Houston, TX, United States; ^6^ Center for Geriatrics and Endocrinology, Sichuan Provincial People’s Hospital, University of Electronic Science and Technology of China, Chengdu, Sichuan, China; ^7^ Center for Critical Care Medicine, Sichuan Provincial People’s Hospital, University of Electronic Science and Technology of China, Chengdu, Sichuan, China; ^8^ Clinical Immunology Translational Medicine Key Laboratory of Sichuan Province, Sichuan Provincial People’s Hospital, University of Electronic Science and Technology of China, Chengdu, Sichuan, China

**Keywords:** regulatory B cells (Bregs), interleukin-10 (IL-10), transforming growth factor-beta (TGF-β), type 1 and type 2 diabetes mellitus (T1DM and T2DM), β-cell autoimmunity, islet β-cell

## Abstract

Regulatory B cells (Bregs) are pivotal modulators of immune tolerance, suppressing inflammation through cytokine secretion and cellular interactions. Their role is particularly significant in inflammatory diseases such as type 1 and type 2 diabetes mellitus (T1DM and T2DM), where immune dysregulation contributes to disease progression. In T1DM, Bregs mitigate β-cell autoimmunity via IL-10 production and FOXP3-mediated pathways, but genetic mutations and dysfunctions in these mechanisms exacerbate autoimmunity. In T2DM, chronic inflammation and metabolic stress impair Breg numbers and function, further fueling insulin resistance. While Bregs play a central role in T1DM by directly preventing β-cell destruction, their role in T2DM is more supportive, modulating inflammation in metabolically stressed tissues. Emerging therapeutic strategies aim to enhance Breg function through IL-10 induction, ex vivo expansion, or targeting Breg-specific pathways using gene-editing and small molecules. Future research should explore Breg heterogeneity, novel markers, and personalized therapies to unlock their full potential. Understanding and leveraging the immune tolerance role of Bregs may offer transformative strategies to inhibit inflammatory diseases like diabetes mellitus.

## 1 Introduction

Regulatory B cells (Bregs) are a specialized subset of B lymphocytes that play a crucial role in maintaining immune tolerance and suppressing excessive inflammatory responses (1). Unlike conventional B cells, which are primarily involved in antigen presentation and antibody production, Bregs exert immunosuppressive functions through cytokine secretion and cellular interactions (2). They are essential for preventing autoimmunity and excessive immune activation, which can lead to chronic inflammatory diseases (3, 4).

Inflammatory diseases such as rheumatoid arthritis (RA), multiple sclerosis (MS), and diabetes mellitus are often associated with dysregulated immune responses (5, 6). In the context of diabetes mellitus, both type 1 diabetes (T1DM) and type 2 diabetes (T2DM) arise from chronic inflammation and immune dysfunction (7). T1DM is an autoimmune disease characterized by the destruction of insulin-producing β-cell in the pancreas, driven by autoreactive T cells and an imbalance in immune tolerance mechanisms (8, 9). T2DM, on the other hand, is primarily a metabolic disorder accompanied by low-grade chronic inflammation, which exacerbates insulin resistance and pancreatic dysfunction (10).

Understanding the role of Bregs in these conditions is critical for developing novel therapeutic strategies. Bregs can suppress autoreactive immune cells in T1DM (11), while also mitigating chronic inflammation in T2DM (3). Through the secretion of anti-inflammatory cytokines such as interleukin-10 (IL-10) and transforming growth factor-beta (TGF-β), as well as their interactions with other immune cells, Bregs contribute to restoring immune homeostasis (12). This review highlights the mechanisms of Breg-mediated immune regulation and their therapeutic potential in diabetes mellitus.

## 2 Bregs and immune tolerance: mechanisms of action

Bregs maintain immune tolerance through a combination of cytokine-mediated suppression, cellular interactions, and pathway regulation (13). These mechanisms allow Bregs to inhibit pro-inflammatory immune responses, control autoimmunity, and protect against tissue damage (3).

### 2.1 Cytokine secretion: suppressing inflammatory responses

Bregs’ immunosuppressive effects are primarily mediated by their secretion of anti-inflammatory cytokines such as IL-10, TGF-β, and IL-35 (14). IL-10, the hallmark cytokine of Bregs, is central to their immune regulatory function, suppressing effector T cell activation and reducing pro-inflammatory cytokine production (15). This is particularly evident in T1DM, where IL-10 inhibits autoreactive T cells targeting pancreatic β-cell (16), and in T2DM, where it mitigates inflammation in adipose tissue, alleviating insulin resistance (10). In experimental diabetic models, transferring IL-10-producing Bregs delays hyperglycemia onset and reduces pancreatic islet inflammation, underscoring IL-10’s protective role (17). Similarly, TGF-β enhances immune tolerance by promoting regulatory T cell (Treg) differentiation and suppressing effector T cell responses (18). By creating a feedback loop with Tregs, TGF-β-producing Bregs further reinforce immune regulation and protect beta cells in T1DM (11).

While IL-10 has garnered much attention, IL-35, another critical cytokine, also plays a significant role in immune suppression. IL-35, produced by Bregs, exerts potent anti-inflammatory effects by inhibiting T cell proliferation and promoting the differentiation of Tregs, thereby enhancing immune tolerance (19). Studies in experimental models of T1DM have shown that IL-35-producing Bregs can reduce pancreatic inflammation and protect β-cell function, similar to IL-10’s effects (20). Clinical studies evaluating IL-35’s therapeutic potential are limited but promising; for example, in patients with autoimmune diseases, such as rheumatoid arthritis, increased IL-35 levels correlate with reduced disease activity and inflammation (21). These findings suggest that IL-35 may serve as a therapeutic target for regulating immune responses in diabetes, offering an additional tool alongside IL-10 to modulate inflammation and preserve β-cell function.

Together, these cytokines enable Bregs to act as a counterbalance to pro-inflammatory immune cells, curbing inflammation in autoimmune and metabolic diseases.

### 2.2 Cellular interactions: regulating T cells and antigen-presenting cells

In addition to cytokine secretion, Bregs interact directly with other immune cells to modulate inflammatory responses. They suppress cytotoxic T lymphocytes (CTLs) and promote Treg expansion through mechanisms, like CTLA-4-mediated interference, with antigen-presenting cells (APCs) (22). This suppressive function is vital in T1DM, where autoreactive T cells drive β-cell destruction (11, 14), and in T2DM, where inflammation exacerbates insulin resistance (23, 24). Bregs also modulate APC function by reducing their expression of MHC class II and co-stimulatory molecules, limiting their ability to activate T cells (25). For instance, Breg-derived IL-10 polarizes macrophages toward an anti-inflammatory M2 phenotype, improving insulin sensitivity in T2DM models (26–28). These cellular interactions highlight the dual role of Bregs in modulating both adaptive and innate immunity to maintain immune tolerance and mitigate disease progression.

### 2.3 Pathway regulation: TLR- and STAT3-mediated Breg activation

The regulatory mechanisms governing Breg activation are equally critical. Toll-like receptor (TLR) and STAT3 signaling pathways are two major regulators of Breg function (13). TLR signaling, particularly through TLR4 stimulation by lipopolysaccharides (LPS), induces IL-10 production in Bregs (29). This pathway is significant in T2DM, where microbial dysbiosis and increased gut permeability elevate circulating LPS levels (30, 31). Most evidence for TLR4-induced IL-10 production comes from murine studies, and similar mechanisms in human Bregs remain less clearly defined. Thus, while the link between microbiota, LPS, and IL-10-producing Bregs is robust in mice, its direct relevance in human immunoregulation remains to be confirmed. By activating Bregs, TLR4 signaling offsets the pro-inflammatory effects of LPS, reducing systemic inflammation and insulin resistance. Similarly, STAT3 signaling is essential for Breg differentiation and function. Cytokines like IL-6 and IL-21 activate STAT3, driving IL-10 production and enhancing Breg-mediated immune suppression (32). Although IL-21 has been shown to activate STAT3 and enhance IL-10 production in murine Bregs, accumulating evidence also suggests a similar role in human Breg biology (29, 33, 34). However, elevated IL-21 levels in T1DM impair Breg function, highlighting the therapeutic potential of targeting STAT3 to restore immune tolerance (35–37). Pharmacological interventions that boost STAT3 activation or TLR signaling in Bregs could enhance their suppressive capacity, offering novel therapeutic approaches for autoimmune and metabolic diseases. Notably, recent studies have identified STAT3 mutations in human patients with early-onset autoimmunity, including T1DM (37). These mutations impair tolerance mechanisms by disrupting CD8+ T cell regulation and potentially affect Breg development. Such findings provide direct evidence that STAT3-mediated pathways are crucial in human immune tolerance.

## 3 Breg dysfunction and diabetes mellitus

Bregs are essential for maintaining immune tolerance, but their dysfunction has emerged as a significant contributor to the pathogenesis of both T1DM and T2DM (11, 24). Despite their distinct etiologies, these diseases share common features of immune dysregulation and chronic inflammation, with impaired Breg-mediated suppression exacerbating disease progression.

### 3.1 Bregs in T1DM

T1DM is an autoimmune disease characterized by the targeted destruction of insulin-producing pancreatic islet β-cells (8). This destruction is driven by autoreactive T cells, which escape immune regulation due to a breakdown in peripheral tolerance. Dysfunctional Bregs in T1DM exhibit reduced IL-10 production, a key anti-inflammatory cytokine critical for suppressing effector T cells and dampening inflammation (38). The diminished IL-10 output allows for the unchecked activation of autoreactive T cells and the promotion of a pro-inflammatory environment within pancreatic islets, accelerating β-cell destruction (39). Genetic factors also contribute to Breg dysfunction in T1DM. Mutations or polymorphisms in genes such as FOXP3, IL10, and CTLA4 impair Breg development and their suppressive capacity. Clinical studies have shown that mutations in FOXP3, a transcription factor essential for regulatory cell function, result in severe autoimmunity, including T1DM. For example, patients with immune dysregulation polyendocrinopathy enteropathy X-linked (IPEX) syndrome, caused by FOXP3 mutations, frequently develop T1DM in early childhood (40, 41). Polymorphisms in the IL10 gene, such as -1082A/G and -592C/A, are associated with reduced IL-10 production in T1DM patients. A study found that carriers of the -1082A allele exhibit significantly lower IL-10 levels, which correlates with diminished Breg function and increased pancreatic inflammation (42). These mutations impair the development and suppressive capacity of regulatory cells, including Bregs, leading to uncontrolled autoreactive T cell activity and β-cell destruction. Similarly, Polymorphisms in CTLA4, such as +49A/G, reduce the ability of Bregs to modulate antigen-presenting cells (APCs), leading to increased autoreactive T cell activation. A meta-analysis involving over 12,000 participants demonstrated that the +49G allele is strongly associated with T1DM susceptibility due to impaired immune regulation (43, 44).

Additionally, polymorphisms in HLA-DR and HLA-DQ genes, which are strongly associated with T1DM susceptibility, have been linked to impaired Breg activity (45, 46). A study analyzing patients with HLA-DR3/DR4 genotypes showed reduced IL-10 production by Bregs, further supporting the role of HLA polymorphisms in the failure to suppress autoreactive T cells (47).

Recent research suggests that Breg survival and function are also influenced by metabolic regulation. Fatty acid oxidation (FAO) and mitochondrial activity are critical for Breg maintenance, as these pathways support energy homeostasis and cytokine production (48). Metabolic stress in T1DM, including altered glucose metabolism and oxidative stress, disrupts FAO, leading to mitochondrial dysfunction in regulatory immune cells, including Bregs. Studies indicate that peroxisome proliferator-activated receptor gamma (PPARγ) activation enhances FAO and promotes Breg survival, while AMPK signaling, a key regulator of cellular energy homeostasis, is essential for maintaining Breg function under metabolic stress (49). Therapeutic strategies aimed at boosting FAO via PPARγ agonists (e.g., pioglitazone) or enhancing AMPK activity (e.g., metformin) may help restore Breg-mediated immune tolerance and slow T1DM progression (50).

Key findings from animal models have demonstrated the importance of Bregs in T1DM. In non-obese diabetic (NOD) mice, depletion of IL-10-producing Bregs accelerates diabetes onset and worsens β-cell destruction, while adoptive transfer of Bregs delays disease progression (11, 51). These studies highlight the critical role of Bregs in maintaining immune tolerance and preventing autoimmune destruction in T1DM.

### 3.2 Bregs in T2DM

In T2DM, a metabolic disorder characterized by insulin resistance and chronic low-grade inflammation, Breg dysfunction occurs against the backdrop of systemic metabolic stress (23, 52). Chronic inflammation in insulin-sensitive tissues, such as adipose tissue and pancreatic islets, reduces the number of IL-10-producing Bregs and impairs their suppressive functions (23). The resulting reduction in Bregs correlates with increased levels of pro-inflammatory cytokines like TNF-α and IL-6, which exacerbate insulin resistance and β-cell stress (53).

Genetic studies also link Breg dysfunction to T2DM pathogenesis. Polymorphisms in the IL-10 gene, such as -592C/A, have been associated with reduced IL-10 production in patients with T2DM (54). A clinical study demonstrated that reduced IL-10 levels in these patients correlate with heightened systemic inflammation, increased insulin resistance, and impaired Breg function (55). Unlike T1DM, where FOXP3 and CTLA4 mutations are more prominent in Breg dysfunction, the role of genetic susceptibility in T2DM is primarily linked to IL10 polymorphisms, reflecting the inflammatory and metabolic nature of the disease. Metabolic stressors, particularly dysregulated lipid metabolism, significantly impact Breg function in T2DM (56, 57). It disrupts pathways essential for their activity, such as PPARγ, AMPK signaling and Toll-like receptor 4 (TLR4) signaling. For example, PPARγ is a transcription factor that regulates lipid metabolism and inflammation. Reduced PPARγ activity impairs IL-10 production in Bregs, weakening their ability to mitigate inflammation in insulin-sensitive tissues (27, 58). Recent studies suggest that targeting AMPK signaling, a crucial regulator of energy metabolism, could mitigate Breg dysfunction in T2DM. AMPK activators like metformin not only improve insulin sensitivity but also promote Breg expansion and IL-10 production in preclinical models (59, 60). Chronic activation of TLR4 by circulating lipopolysaccharides (LPS) in obese individuals drives a pro-inflammatory state, further diminishing Breg-mediated suppression (27). Clinical studies in obese patients with T2DM confirm significantly reduced levels of IL-10^+^ Bregs compared to healthy controls, correlating with heightened systemic inflammation (60). Experimental models have also demonstrated that restoring Bregs improves insulin sensitivity and reduces inflammation in adipose tissue and pancreatic islets, underscoring their potential therapeutic value in T2DM (59, 60).

In summary, the dysfunction of Bregs plays a pivotal role in the immune dysregulation and inflammation underlying both T1DM and T2DM (11, 61). Reduced IL-10 production, genetic susceptibility, and metabolic stress converge to impair Breg-mediated immune tolerance, exacerbating disease progression. These insights highlight the importance of preserving Breg function as a potential strategy to mitigate inflammation and improve outcomes in diabetes mellitus.

## 4 Comparison of Breg roles in T1DM vs. T2DM

Although Bregs play critical roles in both T1DM and T2DM, their contributions differ based on the underlying pathophysiology of each disease. In T1DM, Bregs are directly involved in preventing β-cell autoimmunity, whereas in T2DM, they play a more supportive role in regulating chronic inflammation and metabolic stress (7, 38).

### 4.1 Central role in T1DM

In T1DM, Bregs are central to maintaining immune tolerance and preventing β-cell destruction. Their dysfunction leads to a failure in suppressing autoreactive T cells, resulting in unchecked autoimmunity. Genetic mutations affecting key immune-regulatory genes, such as FOXP3, IL10, and CTLA4, have a profound impact on Breg development and function in T1DM (62, 63). For example, reduced IL-10 production by Bregs directly contributes to pancreatic inflammation and β-cell loss (64). Additionally, the strong association between HLA polymorphisms and Breg dysfunction underscores their critical role in autoimmune diabetes (65). Without effective Breg-mediated tolerance, β-cell autoimmunity progresses rapidly, leading to disease onset.

### 4.2 Supportive role in T2DM

In contrast, Bregs have a more indirect role in T2DM. Rather than preventing autoimmunity, they modulate chronic inflammation and metabolic stress in insulin-sensitive tissues such as adipose tissue and the liver. Dysfunctional Bregs in T2DM are often a consequence of systemic inflammation, metabolic stress, and alterations in pathways such as PPARγ and TLR4 (66, 67). Reduced IL-10 production by Bregs exacerbates inflammation in adipose tissue, contributing to insulin resistance and worsening disease pathology (68). Key studies in obese patients with T2DM have highlighted the link between systemic inflammation and impaired Breg function, emphasizing their importance in controlling metabolic inflammation.

### 4.3 Key differences

The primary distinction between T1DM and T2DM lies in the mechanisms driving Breg dysfunction and their effects on disease progression. In T1DM, Bregs directly regulate the autoimmune response against β-cells, with genetic factors playing a significant role in their dysfunction (11). In T2DM, Bregs primarily act to suppress chronic inflammation, and their dysfunction is driven by external factors such as metabolic stress and environmental triggers (59). These differences highlight the need for tailored therapeutic approaches to restore Breg function in each disease.

In summary, Bregs are indispensable for maintaining immune tolerance, but their dysfunction contributes to the pathogenesis of both T1DM and T2DM. Future research should focus on understanding the unique mechanisms underlying Breg dysfunction in these diseases and developing therapies to enhance their regulatory capacity.

## 5 Therapeutic perspectives

Harnessing the immunosuppressive and immune-regulatory potential of Bregs has emerged as a promising therapeutic strategy, particularly for managing inflammatory diseases such as diabetes mellitus (13). Given the distinct roles and mechanisms of Breg dysfunction in T1DM and T2DM, therapeutic strategies must be tailored accordingly. **Table 1** summarizes key differences in Breg function between T1DM and T2DM, along with corresponding therapeutic strategies aimed at restoring Breg function in each condition.

**Table 1:** Comparison of breg dysfunction and therapeutic strategies in T1DM vs. T2DM.

Features	T1DM	T2DM	Therapeutic Strategies
Primary Role of Bregs	Suppress autoreactive T cells to prevent β-cell destruction	Modulate chronic inflammation in metabolic tissues	IL-10 supplementation, Breg expansion, PPARγ activation
Mechanisms of Dysfunction	Reduced IL-10 production, impaired FOXP3/CTLA-4 signaling, autoreactive T cell activation	Chronic inflammation reduces Breg numbers and IL-10 secretion, alters TLR/PPARγ pathways	Targeting IL-10 and FOXP3 in T1DM, metabolic pathway modulation in T2DM
Genetic Influences	FOXP3, IL-10, and CTLA-4 mutations linked to impaired Breg function	IL-10 polymorphisms, metabolic stress, and altering immune balance	Gene-editing (e.g., CRISPR) for T1DM mutations, metabolic intervention for T2DM
Key Immunological Target	Prevention of β-cell autoimmunity	Reduction of inflammation-driven insulin resistance	IL-10 therapies, TLR modulation, biologics targeting inflammatory cytokines

### 5.1 Enhancing Breg function

In T1DM, where autoimmune destruction of pancreatic islet β-cells drives disease progression, Bregs are pivotal for suppressing autoreactive T cells and dampening inflammatory responses. Strategies to enhance Breg function include the supplementation or induction of IL-10, a key anti-inflammatory cytokine produced by Bregs. Administering recombinant IL-10 or employing agents to stimulate its endogenous production in Bregs could counteract the autoimmunity associated with T1DM (68, 69). Trials involving IL-10 receptor agonists have demonstrated potential for reducing T cell-mediated β-cell destruction (70).

Another promising avenue is the *ex vivo* expansion and reintroduction of Bregs. By isolating Bregs from patients, expanding them *in vitro*, and reintroducing them into the host, immune balance may be restored (71). Advances in cell-culture techniques and cytokine cocktails, such as IL-21 and CpG, have improved the feasibility of this approach. Additionally, modulation of FOXP3 and CTLA4, which are critical for Breg development and suppressive function, offers therapeutic potential (72). Gene-editing tools like CRISPR/Cas9 could facilitate precise corrections of mutations in these genes, thereby restoring Breg-mediated immune tolerance (73).

In T2DM, chronic inflammation driven by metabolic dysregulation and insulin resistance underscores the importance of anti-inflammatory interventions targeting Bregs (74). Strategies to upregulate IL-10 production or modulate TLR-mediated activation in Bregs could alleviate inflammation (3, 29). Specific TLR antagonists have shown promise in preclinical models by enhancing Breg-mediated suppression. Another approach involves activating Bregs through PPARγ agonists. These agents, commonly used as insulin sensitizers, also modulate immune responses (75). Recent studies suggest that PPARγ activation in Bregs enhances their regulatory phenotype, providing dual benefits of improved insulin sensitivity and reduced inflammation (76).

### 5.2 Targeting Breg-related pathways

Emerging gene-editing technologies, such as CRISPR/Cas9, offer transformative possibilities for correcting genetic mutations linked to Breg dysfunction in both T1DM and T2DM. By targeting key genes such as FOXP3, IL10, and CTLA4, these tools could restore Breg function and promote immune tolerance (1, 13). While still in the experimental stage, such approaches represent a significant step toward personalized medicine.

The development of small molecules or biologics to stabilize Breg phenotypes in chronic inflammation also holds promise (77). For instance, small molecules targeting the STAT3 pathway could enhance Breg differentiation and suppress pro-inflammatory responses (78). Monoclonal antibodies against inflammatory cytokines such as TNF-α or IL-6 could indirectly bolster Breg activity by mitigating systemic inflammation (79). These approaches aim to modulate the inflammatory milieu, allowing Bregs to maintain their suppressive function effectively.

Additionally, recent studies suggest that the IL-8-CXCR1/CXCR2 axis plays a role in Breg differentiation. Elevated IL-8 (CXCL8) levels have been observed in inflammatory conditions (80), and its interaction with CXCR1 and CXCR2 receptors influences immune cell function (81). While IL-8 is primarily associated with neutrophil recruitment, emerging evidence indicates that it may contribute to B cell development under certain conditions (82, 83). Given that CXCR1/2 blockade has been shown to reduce inflammation and tissue damage in diabetic complications, targeting this pathway could offer new avenues to enhance Breg differentiation and stability in metabolic disorders (84). Further investigation into the IL-8-CXCR1/CXCR2 axis could provide insights into its role in regulating Bregs and its potential as a therapeutic target in diabetes.

## 6 Future directions

Despite significant progress, several challenges remain in fully understanding and leveraging Bregs for therapeutic purposes. Here, we outline key areas for future research and development:

### 6.1 Addressing Breg heterogeneity

One major hurdle is the heterogeneity of Breg subsets. Current knowledge is limited to a few well-characterized markers, such as CD19^+^CD5^+^CD1d^+^ and CD19^+^TIM-1^+^, but the functional diversity across subsets remains unclear (85, 86). Advanced techniques like single-cell RNA sequencing (scRNA-seq) could elucidate the transcriptional profiles of different Breg subsets, providing insights into their organ-specific roles and responses in diabetes (51). Breg heterogeneity varies significantly between murine and human systems. In mice, well-characterized subsets include CD1dhiCD5+ and CD21hiCD23hi Bregs, while in humans, TIM-1+, CD24hiCD38hi, and CD19+CD25hi Bregs have been identified (13, 32, 87, 88). Functional markers also differ—murine Bregs are primarily IL-10 producers, whereas human Bregs may also utilize IL-35 or TGF-β. These distinctions suggest that translational therapeutic strategies must consider species-specific differences in Breg phenotypes and their regulatory pathways.

### 6.2 Identifying reliable biomarkers

Reliable biomarkers are essential for monitoring Breg function and therapeutic efficacy (89). Identifying novel surface markers or secreted molecules unique to functional Bregs could aid in patient stratification and treatment monitoring. Proteomics and metabolomics approaches may complement genomic studies to uncover these biomarkers.

### 6.3 Developing personalized immunotherapies

Given the genetic and phenotypic variability in Bregs among individuals, personalized immunotherapies tailored to patients’ unique immune profiles hold great promise (90). Integrating omics data with machine learning algorithms could predict the most effective Breg-targeted therapies for individual patients, advancing precision medicine in diabetes care (90).

### 6.4 Exploring organ-specific Breg functions

Understanding how Bregs operate in specific tissues, such as pancreatic islets in T1DM or adipose tissue in T2DM, is crucial for designing targeted therapies. Future research should focus on the local microenvironment’s influence on Breg differentiation and function, using *in vivo* imaging and spatial transcriptomics.

### 6.5 Investigating the Treg-Breg relationship

Bregs and Tregs are two key players for maintaining immune tolerance, yet the extent of their interaction remains incompletely understood. Both cell types exert immunosuppressive effects, with Bregs enhancing Treg differentiation through IL-10 secretion, while Tregs support Breg function via CTLA-4 and IL-2 signaling (23). However, the specific mechanisms governing their reciprocal regulation in diabetes remain unclear (91). Disruptions in this reciprocal regulation may contribute to autoimmune pathology in T1DM and the chronic inflammatory environment in T2DM (3, 91). Future studies should explore how the crosstalk between Tregs and Bregs contributes immune regulation, whether dysregulation leads to uncontrolled immune activation, and how their interaction is influenced by metabolic and inflammatory conditions. A deeper understanding of this relationship could inform novel combination immunotherapies that harness both regulatory cell types for immune modulation in diabetes and other autoimmune diseases.

### 6.6 Developing B cell depletion strategies that spare Bregs

Current B cell-depleting therapies, such as rituximab (anti-CD20), effectively reduce B cell populations but inadvertently eliminate Bregs, potentially worsening immune dysregulation (92, 93). Given the critical immunoregulatory role of Bregs, it is essential to develop strategies that selectively deplete pro-inflammatory B cell subsets while preserving Bregs. While B cell depletion via anti-CD20 therapy such as rituximab has effectively prevented T1DM onset in NOD mice, human clinical trials have yielded modest results. For example, in a pivotal trial by Pescovitz (94), rituximab delayed but did not prevent β-cell decline in recent-onset T1DM. This may be partly due to concomitant depletion of Bregs. Recent strategies combining B cell depletion with Treg enhancement may provide a more balanced approach to restoring immune tolerance.

One promising approach is targeting CD19^+^CD11c^+^ inflammatory B cells, which are enriched in autoimmune diseases and contribute to chronic inflammation (95). Developing bispecific antibodies that simultaneously target CD19 and CD11c could provide a more precise depletion strategy, eliminating pathogenic B cells while sparing IL-10-producing Bregs (96).

Additionally, engineering modified anti-CD19/CD20 monoclonal antibodies with reduced effector functions against Bregs could further refine depletion strategies (97). For example, adjusting the Fc domain to reduce antibody-dependent cellular cytotoxicity (ADCC) and complement-dependent cytotoxicity (CDC) against IL-10^+^ Bregs while maintaining strong depletion of pathogenic B cells could enhance selectivity (98). Other approaches include metabolic or cytokine-based interventions, such as IL-35 or PPARγ agonists, to promote Breg expansion post-depletion (3, 99). Combining selective B cell depletion with Treg-promoting therapies may further ensure immune homeostasis, preventing autoimmunity while maintaining regulatory control.

### 6.7 Advancing cell-based therapies and safety considerations

Cell-based Breg therapies hold great promise, but their scalability, and safety require further investigation. Key areas of focus included developing standardized protocols for Breg isolation, expansion, and reintroduction, while ensuring long-term stability and functionality of Bregs for clinical use. The immunosuppressive properties of Bregs could inadvertently increase the risk of infections, including the reactivation of latent pathogens such as tuberculosis or herpesviruses (100, 101). Additionally, their ability to suppress immune responses could promote a pro-tumorigenic environment, thereby raising concerns about the potential for malignancy (2).

To mitigate these risks, controlled delivery mechanisms are essential. Tissue-targeted Breg therapies or biomaterial scaffolds could localize Breg activity to specific tissues, reducing the risk of systemic immunosuppression and minimizing the chances of infection (102). By confining Breg activity to designated areas, these approaches would help prevent unwanted global immune suppression and limit the possibility of infections caused by broader immunosuppression.

Patient monitoring during and after treatment is equally important for mitigating risks. Rigorous surveillance for signs of infection or tumorigenesis is necessary. Regular assessments of immune function, as well as biomarkers associated with tumor formation, can help identify early signs of adverse effects (103). Early detection of any issues will be critical to ensuring patient safety and enabling timely intervention if necessary.

Additionally, combining Breg-based therapies with adjunctive treatments could help mitigate risks associated with their immunosuppressive nature. For example, co-administering anti-infective agents or immune checkpoint inhibitors could counterbalance the immunosuppressive effects of Bregs and help reduce the risk of infections and malignancy (104). Combining Bregs with other immune-modulating therapies may create a more balanced immune response, enhancing the therapeutic benefit while minimizing safety concerns.

By addressing these safety concerns with a multi-pronged approach, Breg-based therapies can be made safer and more effective, ultimately progressing toward clinical application with minimized risks.

## 7 Conclusions and perspectives

Bregs are indispensable for maintaining immune tolerance and mitigating inflammatory responses. Their roles in both T1DM and T2DM underscore their significance in preserving pancreatic islet integrity and reducing systemic inflammation. In T1DM, Bregs play a central role by directly suppressing autoreactive immune responses and preventing β-cell destruction (16, 17). Conversely, in T2DM, their role is more supportive, modulating chronic inflammation and metabolic dysregulation (74).

Therapeutic strategies centered on Bregs offer immense potential for diabetes treatment. Enhancing Breg function, through IL-10 supplementation, *ex vivo* expansion, or pathway modulation, could significantly improve outcomes in T1DM. In T2DM, targeting inflammation via PPARγ agonists or TLR pathways could alleviate disease progression. Additionally, emerging technologies like CRISPR/Cas9 and single-cell analyses promise to revolutionize our understanding of Breg biology, enabling the development of personalized and precise interventions.

Future research should prioritize addressing Breg heterogeneity, identifying biomarkers, and exploring their organ-specific functions. A deeper understanding of how distinct Breg subsets contribute to immune regulation in T1DM and T2DM will be essential for refining therapeutic strategies. Advances in single-cell technologies and multi-omics approaches could facilitate the identification of disease-specific Breg signatures, aiding in the development of targeted interventions. Additionally, optimizing protocols for *ex vivo* Breg expansion and *in vivo* modulation remains a critical challenge. Investigating how metabolic and inflammatory environments shape Breg function may uncover novel pathways for therapeutic intervention. Moreover, translating gene-editing technologies like CRISPR/Cas9 into clinical applications requires further validation to ensure safety and efficacy.

By integrating these insights with cutting-edge biotechnology, future Breg-targeted therapies could provide personalized and scalable solutions for diabetes and other inflammatory diseases. Addressing these key gaps will be essential for unlocking the full therapeutic potential of Bregs and improving patient outcomes.
